# L-Asparaginase, Doxorubicin, Vincristine, and Prednisolone (LHOP) Chemotherapy as a First-Line Treatment for Dogs with Multicentric Lymphoma

**DOI:** 10.3390/ani11082199

**Published:** 2021-07-26

**Authors:** Jih-Jong Lee, Albert Taiching Liao, Shang-Lin Wang

**Affiliations:** 1 Graduate Institute of Veterinary Clinical Science, School of Veterinary Medicine, National Taiwan University, Taipei 10617, Taiwan; jacklee@ntu.edu.tw; 2 Animal Cancer Center, College of Bioresources and Agriculture, National Taiwan University, Taipei 10617, Taiwan; atliao@ntu.edu.tw; 3 National Taiwan University Veterinary Hospital, College of Bioresources and Agriculture, National Taiwan University, Taipei 10672, Taiwan; 4 Department and Graduate Institute of Veterinary Medicine, School of Veterinary Medicine, National Taiwan University, Taipei 10617, Taiwan

**Keywords:** canine, l-asparaginase, lymphosarcoma, oncology, outcome

## Abstract

**Simple Summary:**

Cyclophosphamide exhibits the weakest therapeutic effect compared with vincristine and doxorubicin in the traditional CHOP chemotherapeutic protocol for the treatment of canine lymphoma in previous studies. We want to evaluate the therapeutic effect of a new multi-drug chemotherapy (LHOP) that uses l-asparaginase in place of cyclophosphamide in the traditional CHOP protocol. Twenty dogs with multicentric lymphoma were treated using the LHOP protocol, and the outcomes were historically compared with those of dogs that received CHOP chemotherapy in the same institution. The adverse effects of l-asparaginase were well tolerated and self-limiting. The dogs that received LHOP chemotherapy had a significantly longer progression-free survival than the dogs that received CHOP chemotherapy. Our study findings thus indicate that the LHOP protocol can be used as a first-line chemotherapeutic protocol in canine multicentric lymphoma.

**Abstract:**

Cyclophosphamide exhibits the weakest therapeutic effect compared with vincristine and doxorubicin in the CHOP (C, cyclophosphamide; H, doxorubicin; O, vincristine; and P, prednisolone) chemotherapeutic protocol for the treatment of canine lymphoma. Twenty dogs with multicentric lymphoma were treated using the LHOP protocol, which used l-asparaginase in place of cyclophosphamide, and the outcomes were historically compared with those of dogs that received CHOP chemotherapy in the same institution. No significant differences were found in age (*p* = 0.107), body weight (*p* = 0.051), sex (*p* = 0.453), clinical stage V (*p* = 1), substage b (*p* = 0.573), T-cell phenotype (*p* = 0.340), overall response (*p* = 1), and hypercalcaemia status (*p* = 1) between the LHOP and CHOP groups. The adverse effects of l-asparaginase were well tolerated and self-limiting. The median PFS (progression-free survival) and median ST (survival time) in the LHOP group were 344 days (range: 28–940 days) and 344 days (range: 70–940 days), respectively. The median PFS and median ST in the CHOP group were 234 days (range: 49–1822 days) and 314 days (range: 50–1822 days), respectively. The dogs that received LHOP chemotherapy had a significantly longer PFS than the dogs that received CHOP chemotherapy (*p* = 0.001). No significant difference was observed in ST between the LHOP and CHOP groups (*p* = 0.131). Our study findings thus indicate that the LHOP protocol can be used as a first-line chemotherapeutic protocol in canine multicentric lymphoma.

## 1. Introduction

Lymphoma is the most common malignant hematopoietic neoplasm in dogs. The annual incidence is reported to be 24 and 114 per 100,000 dogs [1,2]. Numerous chemotherapeutic protocols have been developed to treat canine lymphoma and yield different outcomes. CHOP-based chemotherapeutic protocols (C, cyclophosphamide; H, doxorubicin; O, vincristine; and P, prednisolone) are the most effective treatment, with a reported remission rate of greater than 85% and survival times ranging from 8 to 12 months [3,4,5]. However, evaluating the efficacy of each drug in this multi-drug protocol is difficult. Two studies investigated the efficacy of each drug in dogs with lymphoma under treatment with CHOP protocol and found that cyclophosphamide exhibited the weakest therapeutic effect compared with the other drugs. The number of lymphoma cells in the peripheral blood after the first administration of vincristine, cyclophosphamide, and doxorubicin was decreased in 100%, 51.7%, and 96.3% dogs, respectively, by real-time polymerase chain reaction [6]. Furthermore, dogs with lymphoma relapsed more frequently after the administration of cyclophosphamide than of vincristine and doxorubicin [7]. Therefore, the therapeutic outcome might be improved by replacing cyclophosphamide with other cytotoxic drugs in CHOP-based chemotherapy.

L-asparaginase is an enzyme derived from *Escherichia coli* and is commonly used in the treatment of canine lymphoma [8]. Asparagine is required for intracellular protein synthesis. It can be produced within a cell by asparagine synthetase or be absorbed into the cell from the extracellular space. Lymphoma cells are often deficient in asparagine synthetase; therefore, they have an obligate requirement for extracellular asparagine to meet their metabolic needs and maintain cell viability [4]. L-asparaginase depletes systemic asparagine, thereby interfering with protein synthesis and inducing lymphoma cell death [4,8,9].

The aim of this study was to evaluate the response rate, progression-free survival (PFS), and survival time (ST) in a new chemotherapeutic protocol (LHOP protocol) using l-asparaginase in place of cyclophosphamide in the traditional CHOP protocol for the treatment of canine multicentric lymphoma. The hypothesis is that LHOP chemotherapy can produce a better therapeutic outcome than CHOP chemotherapy in canine lymphoma.

## 2. Materials and Methods

### 2.1. Patient Selection and Evaluation

Dogs diagnosed with multicentric large cell lymphoma (according to cytology or histopathology) at the National Taiwan University Veterinary Hospital between September 2018 and October 2020 were enrolled in this study. The study was approved by the Institutional Animal Care and Use Committee of the National Taiwan University (Approval number: NTU107-EL-00079). Dogs who had previously received chemotherapy, corticosteroids, or those without owner approval to join this prospective study were excluded.

We collected data on breed, age, body weight, sex and neuter status, clinical stage and substage, immunophenotype, total calcium concentration, thoracic and abdominal radiographs, abdominal ultrasound, treatment response, dates of therapy start, disease progression, final follow-up, and death. The World Health Organization (WHO) staging system was used to categorise the clinical stage and the substage [10]. The peripheral blood smears of all dogs were evaluated by clinical pathologists in the department of clinical pathology. Bone marrow examination was not routinely performed unless the complete blood cell count showed lymphocytosis or lymphoblasts observed on the peripheral blood smear, indicating bone marrow involvement. The cell surface marker CD34 was examined to distinguish stage V lymphoma from acute lymphocytic leukaemia. The immunophenotype was determined by flow cytometry or immunohistochemistry.

### 2.2. Treatment Protocol

The enrolled dogs received the 19-week LHOP protocol (Table 1). Treatment was delayed 1 week if neutropenia (<3000 cells/μL) or thrombocytopenia (<100,000 cells/μL) was identified in the pre-treatment assessment, or if a clinical condition indicated that chemotherapy was contraindicated. Diphenhydramine was administered subcutaneously at a dose of 1 mg/kg 30 min before l-asparaginase administration.

The follow-up evaluation interval after induction chemotherapy was finished was once monthly during the first 3 months and then every 3 months. The owners were informed that they could visit the clinic anytime when disease progression was observed.

LHOP chemotherapy was administered again when lymphoma relapsed after the first complete LHOP induction chemotherapy, except the doxorubicin was replaced with mitoxantrone (5 mg/m^2^). Rescue therapy was administered with lomustine (60 mg/m^2^) if the tumour relapsed during the initial induction chemotherapy.

### 2.3. Response Assessment

The therapeutic response was determined using the Veterinary Cooperative Oncology Group response evaluation criteria for lymphoma [11]. A complete response (CR) was characterised by the disappearance of all measurable diseases; a partial response (PR) was characterised by a decrease (>30% but <100%) in the mean sum longest diameter of target lesions; stable disease (SD) was characterised by a <30% decrease or <20% increase in target lesions compared to baseline; and progressive disease (PD) was characterised by a >20% increase in target lesions or the development of a new lesion.

The PFS was calculated from the initiation of treatment to the time of disease progression. The ST was calculated from the initiation of treatment to the time of patient death. Dogs were censored in remission duration analysis for the following reasons: (1) relapse had not occurred before the end of the study period; (2) lost to follow-up during remission; or (3) death before relapse. Dogs were censored in survival analysis for the following reasons: (1) lost to follow-up; (2) death from causes other than lymphoma; or (3) they were still alive.

### 2.4. Toxicity

The major adverse effects of l-asparaginase were hypersensitivity reactions and the decrease in protein synthesis. Hypersensitivity reactions can occur with clinical signs of pruritus, vomiting, and collapse. Pancreatitis or other gastrointestinal disturbances may be noted and associated with asparaginase’s effects on protein synthesis [12]. The dogs were monitored for allergic reactions after l-asparaginase administration in the clinic for 30 min. Gastrointestinal toxicities were recorded by the owner at home and graded according to the Veterinary Cooperative Oncology Group Common Terminology Criteria for Adverse Events by the veterinarian [13]. The follow-up complete blood cell count was examined and recorded 1 week later.

### 2.5. Historical Comparison Group That Received CHOP Chemotherapy Protocol

Dogs with multicentric lymphoma that received the CHOP chemotherapy protocol in the same institution between June 2010 and May 2018 were retrospectively reviewed and compared with the dogs that received the LHOP protocol between September 2018 and October 2020. The sequence order and dosage of the cytotoxic drugs were the same between the CHOP protocol and LHOP protocol except for the usage of cyclophosphamide or l-asparaginase. Information on breed, sex and neuter status, age, body weight, clinical stage and substage, immunophenotype, total calcium concentration, treatment response, dates of therapy start, disease progression, final follow-up, and death were reviewed and analysed.

### 2.6. Statistical Analysis

The age and body weight of canine lymphoma patients in the LHOP and CHOP treatment groups were compared using the Student’s *t*-test. Sex, clinical stage and substage, immunophenotype, hypercalcaemia, and response rate were analysed using Fisher’s exact test. The median PFS and ST were determined using the Kaplan–Meier analysis, and the differences between the two groups were assessed using the log-rank test. A *p*-value of <0.05 was considered statistically significant. Statistical calculations were performed using a commercial statistical software package (IBM SPSS Statistics version 21).

## 3. Results

### 3.1. LHOP Protocol

Twenty canine multicentric lymphoma patients received the LHOP chemotherapeutic protocol in the study. The dogs were mixed breed (*n* = 7), Maltese Terriers (*n* = 3), Chihuahuas (*n* = 2), and one each of Beagle, Welsh Corgi, Dachshund, French Bulldog, Golden Retriever, Standard Poodle, Pug, and Yorkshire Terrier. The median age and median body weight were 9.5 years (range: 4–14 years) and 9.6 kg (range: 2.2–37.7 kg). There were 8 male dogs (5 intact) and 12 female dogs (2 intact). A total of 3, 15, and 2 dogs were diagnosed with WHO stage III, stage IV, and stage V lymphomas, respectively. Thirteen and seven dogs were classified as substage a and substage b, respectively. Seventeen and three dogs were diagnosed with B-cell and T-cell lymphomas, respectively. Hypercalcemia was not diagnosed in any dog before receiving the LHOP protocol. 

Eighteen dogs exhibited CR, and two dogs exhibited PR. Eleven and one dogs were censored in the PFS analysis because they did not relapse before the end of the study and died before relapse, respectively. Four and eight dogs were censored in the ST analysis due to death from causes other than lymphoma and still being alive at analysis, respectively. The median PFS and median ST were 344 days (range: 28–940 days) and 344 days (range: 70–940 days), respectively. 

Eighty-two cycles of l-asparaginase administration were conducted on 20 dogs in this study. Adverse events included 13 (15.9%), 20 (24.4%), 3 (3.7%), and 4 (4.9%) of lethargy, anorexia, vomiting, and diarrhoea, respectively. Five dogs (25%) did not experience any adverse event. Most adverse events were grade 1 and were self-limiting. No allergic reactions or chemotherapy-induced neutropenia were observed by the owners or the veterinarian (Table 2).

### 3.2. CHOP Protocol

Sixty-nine canine multicentric lymphoma patients that received the CHOP chemotherapeutic protocol were retrospectively reviewed and analysed. The dogs were mixed breed (*n* = 20), Golden Retrievers (*n* = 13), Welsh Corgis (*n* = 6), Maltese Terriers (*n* = 5), Beagles (*n* = 5), Miniature Schnauzers (*n* = 5), Shih Tzus (*n* = 3), Dachshunds (*n* = 2), Siberian huskies (*n* = 2), Yorkshire Terriers (*n* = 2), and one each of Border Collie, Cavalier King Charles Spaniel, Cocker Spaniel, Pomeranian, Toy Poodle, and West Highland White Terrier. The median age and median body weight were 8 years (range: 2–14 years) and 14 kg (range: 1.9–39.9 kg). There were 35 male dogs (19 intact) and 34 female dogs (9 intact). A total of 1, 25, 36, and 7 dogs were diagnosed with WHO stage II, stage III, stage IV, and stage V lymphomas, respectively. A total of 51 and 18 dogs were classified as substage a and substage b. Immunophenotyping was examined in 51 dogs, including 48 B-cell and 3 T-cell lymphomas. One dog was diagnosed with hypercalcaemia before receiving the CHOP protocol. 

In total, 61 dogs exhibited CR, 5 dogs exhibited PR, and 3 dogs exhibited SD. Six and two dogs were censored in the PFS analysis because the dogs were lost to follow-up during remission and died before relapse, respectively. Twelve and four dogs were censored in the ST analysis because the dogs were lost to follow-up and died from other causes, respectively. The median PFS and median ST were 234 days (range: 49–1822 days) and 314 days (range: 50–1822 days), respectively. 

### 3.3. Comparison between the LHOP and CHOP Groups

**Figure 1:**
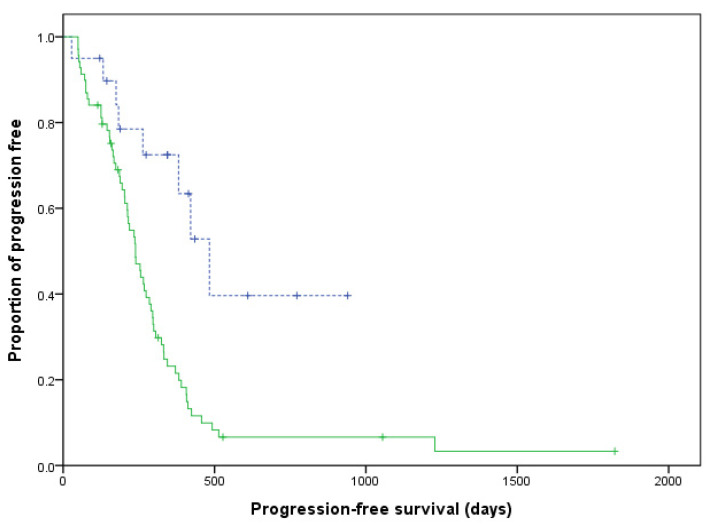
Kaplan–Meier curves of the progression-free survival duration for dogs with lymphoma. Dogs that received the LHOP chemotherapy are indicated by a dashed line, whereas dogs that received the CHOP chemotherapy are indicated by a solid line (*p* = 0.001). Tick marks indicate censored patients.

**Figure 2:**
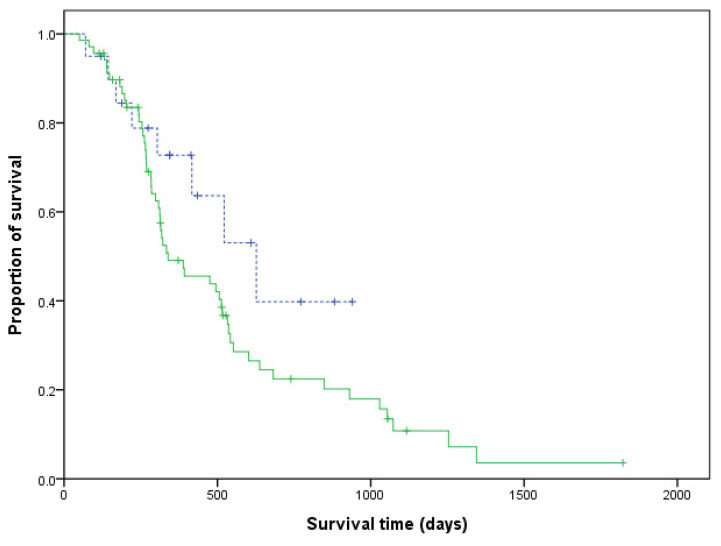
Kaplan–Meier curves of the survival time for dogs with lymphoma. Dogs that received the LHOP chemotherapy are indicated by a dashed line, whereas dogs that received the CHOP chemotherapy are indicated by a solid line (*p* = 0.131). Tick marks indicate censored patients.

No significant differences were found in age (*p* = 0.107), body weight (*p* = 0.051), sex (*p* = 0.453), clinical stage V (*p* = 1), substage b (*p* = 0.573), T-cell phenotype (*p* = 0.340), overall response (*p* = 1), and hypercalcaemia status (*p* = 1) between the LHOP and CHOP groups (Table 3). The dogs that received the LHOP chemotherapy had a significantly longer PFS than the dogs under the CHOP chemotherapy (*p* = 0.001; Figure 1). No significant difference was found in ST between the two groups (*p* = 0.131; Figure 2).

## 4. Discussion

Our results show that the PFS of dogs with lymphoma that received the LHOP chemotherapy was significantly longer than the PFS of those that received the traditional CHOP chemotherapy. A possible explanation could be found in the cytotoxic effects at the different phases of the cell cycle. The basis of chemotherapeutic drug activity is the targeting of dividing cells through the interference with processes involved in progression through the cell cycle. The major classes of cytotoxic drugs work at various steps in the processes of DNA replication (S phase of the cell cycle) and subsequent cell division (M phase) [14]. The components of the traditionally used CHOP chemotherapy protocol are vincristine, cyclophosphamide, doxorubicin, and prednisolone. Prednisolone interacts with the glucocorticoid receptor, thereby inducing the apoptosis of hematopoietic tumour cells [15,16]. However, the mechanisms of apoptosis induction are still not completely understood. Vincristine is a vinca alkaloid that binds to tubulin and inhibits microtubule assembly. It disrupts the mitotic spindle apparatus (M phase) and causes metaphase arrest and cytotoxicity [17]. Cyclophosphamide is an alkylating agent that acts by cross-linking DNA double strands and interferes with DNA replication (S phase) [18]. Doxorubicin is an antitumour antibiotic with a multi-modal mechanism of cellular toxicity that includes the inhibition of RNA and DNA polymerases and topoisomerase II, DNA intercalation and alkylation, and reactive oxygen species generation [19,20,21]. Although doxorubicin is thought to be cell-cycle non-specific, it functions most effectively during the S phase [22]. Cyclophosphamide and doxorubicin predominantly function in the S phase and may have overlapping therapeutic effects on tumour cells. L-asparaginase is an enzymatic antineoplastic agent that acts by depleting extracellular asparagine, resulting in the inhibition of protein synthesis and eventual cell apoptosis [4]. L-asparaginase is mostly active in the G1 phase and may have a synergic effect with doxorubicin and vincristine, thereby inducing more lymphoma cell deaths and conferring longer remission times [23,24].

No significant difference was observed in ST between the LHOP and CHOP groups. However, eight dogs (40%) were still alive at the end of the study and were censored in the ST analysis, which might render the result less significant. Among these eight dogs alive, seven dogs were B-cell lymphoma, and one dog was T-cell lymphoma. There were one stage IIIa, four stage IVa, two stage IVb, and one stage Vb lymphoma according to WHO clinical staging. According to our previous experience in treating lymphoma dogs, many owners choose a less aggressive treatment at the tumour relapse, even without drug resistance to the therapy, which may shorten the ST. Therefore, prolonging the first remission duration should be attempted to provide better tumour control. The result of our study indicated that the LHOP chemotherapy exhibited a significantly longer PFS and should be considered as a first-line treatment protocol for dogs with multicentric lymphoma.

Cyclophosphamide was replaced by l-asparaginase in the LHOP chemotherapy and can be held and used as a rescue agent. The primary goal for the treatment of relapsed lymphoma is palliation [13]. Owners may prefer oral medication at home rather than an injective formulation at the hospital when the tumour relapses. Therefore, the oral form of cyclophosphamide can be easily given at home and provides equal amounts of active metabolites and therapeutic effects with the intravenous form for treating relapsed dogs [25].

When a multi-drug chemotherapy protocol is designed, each of the drugs should be effective as a single agent, they should not be cross-resistant, and they ideally should exhibit no overlapping toxicities [12]. L-asparaginase is already known to exhibit a cytotoxic effect on canine lymphoma [8,26]. Unlike other chemotherapeutic drugs, it is not influenced by p-glycoprotein to transport the cytotoxic agent from intracellular to extracellular space [27]. The major side effect of l-asparaginase is allergic reaction, which usually occurs within 60 min but might appear as late as 4–6 h post-administration [8,28]. However, no allergic reactions were observed in any of the 82 injection episodes in the present study. Furthermore, the administration of diphenhydramine 30 min before l-asparaginase administration can minimize this side effect. Pancreatitis is another adverse effect related to l-asparaginase. This concern is mostly based on human data and on sporadic case reports in the veterinary literature [29]. However, recent veterinary studies report the incidence of l-asparaginase-associated pancreatitis to be extremely low [28,29]. We did not routinely evaluate canine pancreatic lipase immunoreactivity in the dogs with gastrointestinal upset after l-asparaginase administration. The most adverse gastrointestinal event in our study was anorexia, followed by lethargy, diarrhoea, and vomiting. These events were mostly grade 1 (82.5%) and were all self-limiting without further medication. No neutropenic episodes were observed after 1 week of l-asparaginase administration, which indicates it is safe to use with other chemotherapeutic drugs that have bone marrow toxicity [8,12]. Given the advantages listed above, l-asparaginase is a good candidate component in a multi-drug chemotherapy protocol.

The treatment interval between two l-asparaginase administrations in the LHOP protocol was 5 weeks, which was longer than those in other protocols such as l-asparaginase, lomustine, and prednisone rescue protocols [12]. A longer treatment interval between two l-asparaginase administrations may decrease the possibility of overlapping toxicity of the drug.

The other major adverse concern about l-asparaginase administration is the formation of antibodies and the development of drug resistance. Antibodies potentially could reduce or neutralize asparaginase activity in different ways, including the increased clearance by opsonization and the binding of Ig-drug complexes to Fc receptors and the direct inhibition by steric hindrance or by the binding to the proper catalytic site [30]. In a previous study, the average antibody prevalence was 30% (3/10) in dogs treated with single l-asparaginase, and the prevalence increased to 57% (4/7) after two l-asparaginase injections [30]. However, the route of administration could affect the frequency of antibody formation [30]. The dogs received subcutaneous injections of l-asparaginase in that study, which was different from our intramuscular route. L-asparaginase can be administered subcutaneously or intramuscularly. Valerius et al. found that the intramuscular injection of l-asparaginase can result in a faster response to chemotherapy, a longer remission time, and a longer ST in dogs with lymphoma than subcutaneous injection [31]. To produce a better outcome, we used intramuscular injection in all l-asparaginase treatments. Identifying the route of administration that is less immunogenic is important not only for preventing clinical hypersensitivity but also for preserving tumour responsiveness to the drug by diminishing antibody formation [30]. Further study is needed to clarify the outcome and antibody formation between different administration routes.

The incidence of T-cell lymphoma in this study is low. A possible explanation is that T-cell lymphoma has a worse outcome than B-cell lymphoma, and the owner was more inclined to refuse further treatment than to join our clinical trial. T-cell lymphoma is more commonly associated with paraneoplastic hypercalcaemia [32]. Corticosteroid was often administrated at the first-line clinic for treating emergency hypercalcaemia before being referred to our hospital. Therefore, many T-cell lymphoma dogs did not fit our inclusion criteria and were excluded from our study.

This study had some limitations. The number of lymphoma dogs in this prospective study is small; a larger study may be needed to reconfirm our results. The rescue protocol used in the CHOP group could not be standardized since the cases were retrospectively reviewed. Forty percent of dogs were still alive at the end of the study and were censored in the ST analysis. Therefore, the statistical difference obtained in ST between the two treatment groups may have been affected.

## 5. Conclusions

Our study findings show that the LHOP chemotherapeutic protocol provides a longer PFS and similar ST when compared with the historical CHOP protocol in treating canine multicentric lymphoma. Furthermore, the adverse effects of l-asparaginase were well tolerated and self-limiting. Therefore, the LHOP protocol can be used as a first-line chemotherapeutic treatment in canine multicentric lymphoma.

## Figures and Tables

**Table 1:** LHOP chemotherapy protocol.

	Week																
	1	2	3	4	5	6	7	8	9	11	12	13	14	16	17	18	19
Vincristine (0.7 mg/m^2^ IV)	**˙**		**˙**			**˙**		**˙**		**˙**		**˙**		**˙**		**˙**	
L-asparaginase (400 U/kg IM)		**˙**					**˙**				**˙**				**˙**		
Doxorubicin (30 mg/m^2^ IV)				**˙**					**˙**				**˙**				**˙**
Prednisolone (PO q24 h)	2 mg/kg × 7 d, then 1.5 mg/kg × 7 d, then 1.0 mg/kg × 7 d, then 0.5 mg/kg × 7 d, then stop																

**Table 2:** Adverse events of l-asparaginase administration.

		Adverse Events			
Dog	Injection Times	Lethargy	Anorexia	Vomiting	Diarrhea
1	4	1 (Grade 1)	1 (Grade 1) 1 (Grade 2)	-	-
2	4	-	-	-	-
3	4	-	1 (Grade 1) 1 (Grade 2)	-	-
4	6	-	1 (Grade 1)	-	-
5	4	-	1 (Grade 1)	-	-
6	4	1 (Grade 1)	1 (Grade 1)	-	-
7	4	-	1 (Grade 1)	-	-
8	8	1 (Grade 1)	2 (Grade 1)	-	-
9	2	-	-	-	-
10	2	-	-	-	-
11	4	2 (Grade 1)	2 (Grade 1)	-	1 (Grade 1) 1 (Grade 2)
12	4	1 (Grade 1)	1 (Grade 1)	-	1 (Grade 1)
13	4	2 (Grade 1)	1 (Grade 2)	2 (Grade 1) 1 (Grade 2)	1 (Grade 1)
14	4	-	1 (Grade 1)	-	-
15	4	-	-	-	-
16	4	1 (Grade 1)	1 (Grade 1) 1 (Grade 2)	-	-
17	4	-	-	-	-
18	1	1 (Grade 1)	-	-	-
19	3	1 (Grade 1)	1 (Grade 1)	-	-
20	8	2 (Grade 1)	1 (Grade 1) 1 (Grade 2)	-	-
Total	82	13 (Grade 1)	15 (Grade 1) 5 (Grade 2)	2 (Grade 1) 1 (Grade 2)	3 (Grade 1) 1 (Grade 2)

**Table 3:** Comparisons on signalment, negative prognostic factors, response, progression-free survival, and survival time between the LHOP and the CHOP treatment groups.

	LHOP (*n* = 20)	CHOP (*n* = 69)	*p*-Value
Age (years)	9.5	8.2	0.107
Body weight (kg)	11.3	16	0.051
Sex			
Female	12 (60%)	34 (49.3%)	0.453
Male	8 (40%)	35 (50.7%)	
Clinical stage V	2 (10%)	7 (10.1%)	1
Substage b	7 (35%)	18 (26.1%)	0.573
T cell	3 (15%, 3/20)	3 (5.9%, 3/51)	0.340
Hypercalcaemia	0 (0%)	1 (1.4%)	1
Overall response (CR + PR)	20 (100%)	66 (95.7%)	1
Median progression free survival (days)	344	234	0.001
Median survival time (days)	344	314	0.131

## Data Availability

The data that support the findings of this study are available from the corresponding author upon reasonable request.
